# The function of the Arabidopsis receptor kinase THESEUS1 in plant cell wall integrity maintenance: from evolutionary origin to future perspectives

**DOI:** 10.1111/tpj.70701

**Published:** 2026-01-01

**Authors:** Steven T. W. Zwartkruis, Martijn L. Vandegehuchte, Thorsten Hamann

**Affiliations:** Department of Biology, Norwegian University of Science and Technology, Trondheim 7491, Norway

## Abstract

Plants actively monitor the state of their cell walls and adapt their structure and composition as needed. THESEUS1 (THE1), a receptor kinase from the *Catharanthus roseus* RECEPTOR-LIKE KINASE 1-LIKE (*Cr*RLK1L) family, was the first receptor kinase described to be involved in this maintenance of cell wall integrity in *Arabidopsis thaliana*. It contributes to the regulation of cell wall stiffness, participates in lateral root development, and modulates production of the stress hormones abscisic acid and jasmonic acid. Besides, it is required for responses to cellulose biosynthesis inhibition, such as growth inhibition and ectopic lignification. The THE1 ligand, RAPID ALKALINIZATION FACTOR 34, and one intracellular interaction partner, GUANINE EXCHANGE FACTOR 4, have been identified. However, relatively little is known about other interaction partners and mechanisms by which THE1 influences downstream responses. Given that FERONIA, a related member of the *Cr*RLK1L family, has dozens of interaction partners it is likely that there is a wealth of interactors waiting to be described for THE1. Here, we shed light on the evolutionary origin of THE1 and describe the many open questions surrounding THE1-dependent signaling processes. We expect that many of these questions will be answered in the coming years and that these answers will provide more insight into the molecular mechanism of cell wall integrity maintenance mediated by THE1.

## Introduction

Plant cells are enclosed by a rigid yet dynamic carbohydrate-rich matrix known as the plant cell wall. The plant cell wall confers biotic- and abiotic stress tolerance, provides structural support to the plant as a whole, and determines cell shape (Cosgrove, 2024b; Delmer et al., 2024). Environmental stresses, such as salt stress, pathogen infections, and mechanical damage, alter the chemical and physical properties of the cell wall (Novaković et al., 2018; Munzert & Engelsdorf, 2025). The state of the cell wall is sensed by molecular components of the plant cell, which in turn activate adaptive responses including alteration of cell wall structure or composition in a process known as cell wall integrity maintenance (CWI) (Wolf, 2022; Baez et al., 2022). The importance of cell wall structure and composition is highlighted by the large number of cell wall mutants with altered tolerance to biotic and abiotic stresses (Baez et al., 2022; Munzert & Engelsdorf, 2025). However, how exactly mechanical and chemical cues are perceived and translated into physiological responses is not yet fully understood (Bacete & Hamann, 2020; Wolf, 2022).

Receptor kinases (RKs) are plasma membrane-localized proteins with an ectodomain enabling signal perception and an internal serine/threonine kinase domain capable of phosphorylating intracellular signaling components, making RKs prime candidates for transducing information about the state of the cell wall to the cell interior (Engelsdorf & Hamann, 2014). Over 610 *RKs* are encoded in the genome of *Arabidopsis thaliana* (hereafter Arabidopsis), and *RKs* are highly abundant in other plant genomes as well (Shiu & Bleecker, 2001; Ngou et al., 2024). Among the RKs, the *Catharanthus roseus* RECEPTOR-LIKE KINASE 1-LIKE (*Cr*RLK1L) family is of particular importance in determining responses to cell wall derived cues. The *Cr*RLK1L family contains 17 members in Arabidopsis, which play a role in reproduction, growth, and adaptation to environmental stress, reflecting the wide range of processes for which control of CWI is crucial (Lindner et al., 2012; Malivert & Hamant, 2023; Cheung, 2024; Tang & Guo, 2025; Manchanda & Geitmann, 2025). Small secreted peptides of the RAPID ALKALINIZATION FACTOR (RALF) family are ligands of *Cr*RLK1L members (Haruta et al., 2014; Xiao et al., 2019; Abarca et al., 2021). THESEUS1 (THE1) is the first member of the *Cr*RLK1L family identified in Arabidopsis and was found in a mutant suppressor screen of the cellulose deficient mutant *procuste* (*prc1-1*) (Desnos et al., 1996; Fagard et al., 2000; Hématy et al., 2007). It is also required for the response to pharmacological inhibition of cellulose production by the cellulose biosynthesis inhibitor isoxaben (ISX) (Scheible et al., 2001; Denness et al., 2011; Merz et al., 2017). More recently, roles for THE1 in modulating the plant hormones abscisic acid (ABA) and jasmonic acid (JA) have been revealed (Engelsdorf et al., 2018; Bacete et al., 2022). Most THE1-dependent phenotypes only become apparent upon impairment of CWI (Hématy et al., 2007). THE1 was implicated in the response to the fungal pathogen *Botrytis cinerea* (Qu et al., 2017). Currently, FERONIA (FER), another *Cr*RLK1L, is the best-studied member of the family. It is required for a plethora of processes, ranging from immunity to development (Malivert & Hamant, 2023; Cheung, 2024).

In this review, we explore what distinguishes THE1 from other *Cr*RLK1L family members and investigate its evolutionary origins. We also examine current knowledge about THE1 considering key processes in cell signaling. Although significant progress has been made in understanding THE1, including the identification of a ligand and the characterization of mutant phenotypes, important gaps remain in our understanding of how THE1-dependent mutant phenotypes arise. Based on progress in research on related RKs we speculate about possible components of THE1-dependent signaling, which remain to be elucidated. Lastly, we highlight which novel techniques and methodologies will enable us to fill the gaps in our knowledge about THE1 in the future.

## Protein structure and post-translational regulation

**Figure 1:**
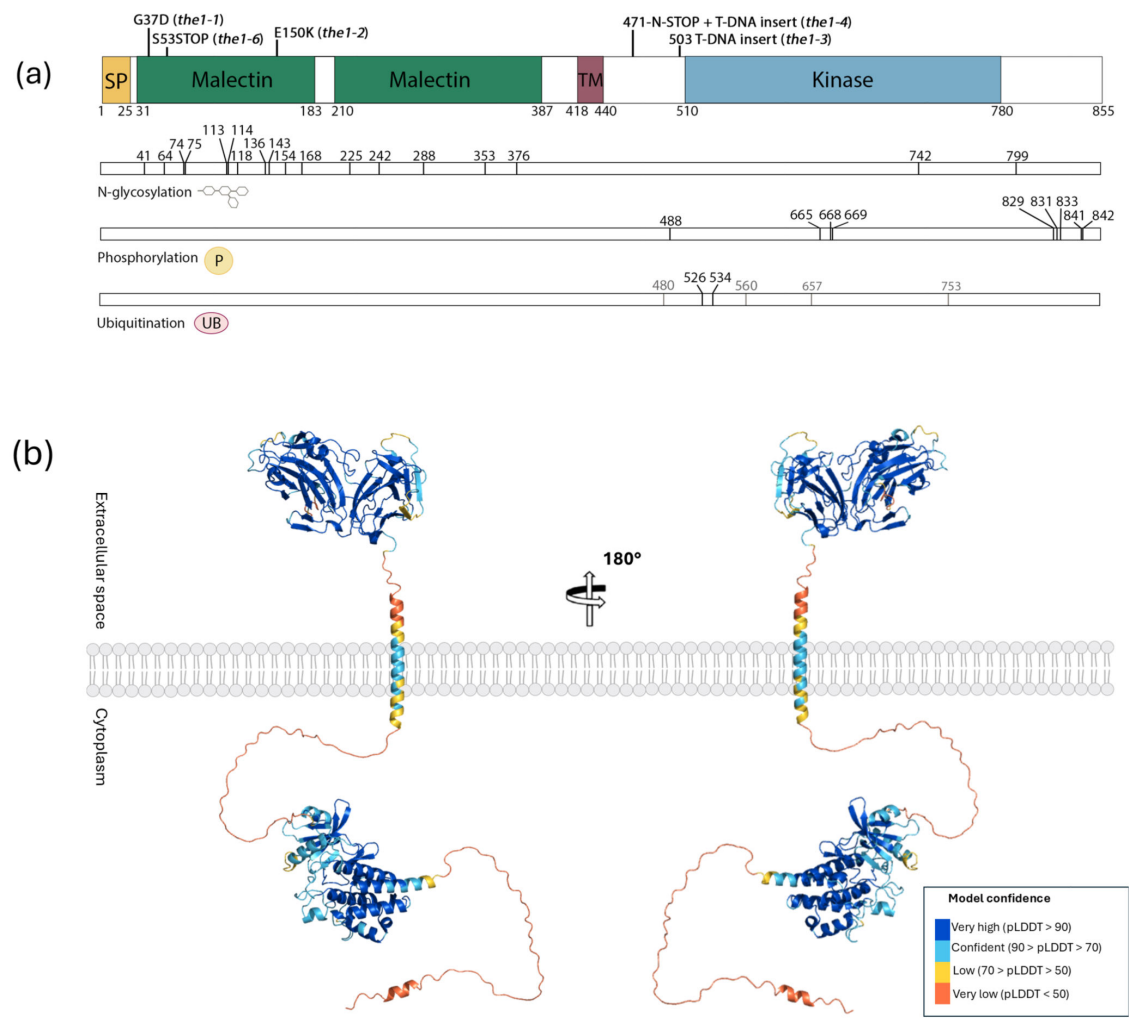
Composition and structure of THESEUS1 (THE1). (a) Overview of the THE1 protein structure and post-translational modifications. The mutant alleles are indicated: *the1-1, the1-2, the1-3* and *the1-6* are loss-of-function mutants, and *the1-4* is a hypermorphic allele. Potential N-glycosylation sites are indicated based on the presence of the N-X-S/T motif. Phosphorylation sites are shown based on data from the PhosPhAt 4.0 database (Table S1) (Heazlewood et al., 2008). Ubiquitination sites are indicated based on observations by Grubb et al. (2021). Ubiquitination sites in black are backed by more observations than the sites in grey. (b) 3D models of THE1 predicted in AlphaFold. The predicted structure is colored by pLDDT score, with higher values indicating stronger confidence in model accuracy. The model was modified in PyMOL V3.2 by rotating bonds in regions with low folding confidence values to allow better visualization of the structure.

THE1 consists of an extracellular part with two malectin-like domains, a transmembrane domain, and an intracellular serine/threonine kinase domain (Figure 1a, b). Malectin domains were first described in the frog species *Xenopus laevis* as di-glucose binding domains (Schallus et al., 2008). However, the *Cr*RLK1L malectin-like domains only show limited similarity to this animal malectin domain (Boisson-Dernier et al., 2011; Moussu et al., 2018). Instead the malectin-like domain of the related *Cr*RLK1Ls, FER, BUDDHAS PAPER SEAL1 (BUPS1), and ANXUR1/2 (ANX1/2) were shown to bind to pectin *in vitro* (Feng et al., 2018; Lin et al., 2022). A short extracellular juxtamembrane region connects the extracellular part of the protein to a transmembrane helix. An intracellular juxtamembrane region connects the transmembrane helix to a serine/threonine kinase domain which has autophosphorylation activity *in vitro* (Hématy et al., 2007), indicating that the kinase is catalytically active. Lastly, THE1 has a C-terminal tail, which may be a target for regulatory post-translational modifications (Bender & Zipfel, 2023).

THE1 is expected to undergo several regulatory post-translational modifications. For example, the extracellular region of THE1 contains 16 N-glycosylation motifs (N-X-S/T)(Figure 1a), which remain to be experimentally validated. The validation of these sites may be relevant because N-glycosylations are important for protein folding, protect the ectodomain from degradation in the apoplast, or play a role in ligand binding and complex formation (Häweker et al., 2010; Nagashima et al., 2018; Jia et al., 2024). Generally, RKs are active when their kinase loop is phosphorylated (Nolen et al., 2004; Bender & Zipfel, 2023). Three phosphorylation sites are located in the activation loop of the kinase domain at residues T665, T669, and S668 according to data from the PhosPhAt 4.0 Arabidopsis protein phosphorylation site database (Figure 1a, Table S1) (Heazlewood et al., 2008). Dephosphorylation of these phosphorylation sites in the kinase loop by a phosphatase can be a way to turn down the activity of THE1. For example, the clade H phosphatase PP2C12 was described to negatively regulate FER by dephosphorylating T696 located in the activation loop of FER (Hou et al., 2025). The C-terminal tail of THE1 is phosphorylated in two mutually exclusive ways. The phosphorylation occurs either at one or more of the S829, T831 and T833 residues; or only at T841 and T842 (Table S1) (Hématy et al., 2007). Residue S488, located in the region between the transmembrane domain and the kinase domain, represents another predicted phosphosite (Figure 1a, table S1). Phospho-null and phospho-mimetic variants of THE1 are needed to study the role of phosphorylation in the regulation of the activity of THE1. Ubiquitination and phosphorylation can target RKs for clathrin-mediated endocytosis (Claus et al., 2018). STRUBBELIG (SUB), another RK implicated in CWI maintenance and the response to cellulose biosynthesis inhibition, is ubiquitinated and undergoes clathrin-mediated endocytosis (Gao et al., 2019). Treatment of FER with its RALF1 ligand induces receptor internalization, providing evidence that ligand-dependent internalization occurs in the *Cr*RLK1L family (Rößling et al., 2024). A putative ubiquitin ligase named *XB3 ORTHOLOG 4 IN ARABIDOPSIS THALIANA* was found to be upregulated after RALF34 treatment in wildtype but not in the *the1-1* mutant (Gonneau et al., 2018). This may indicate that THE1 initiates signal termination by transcriptionally regulating ubiquitin ligases, but further experimental validation is required. Six ubiquitination sites have been described for THE1, occurring at residues K480, K526, K534, K560, K657 and K753, with residues K526 and K534 having the highest confidence value (Figure 1a) (Grubb et al., 2021). Both residues are located in the N-terminal region of the kinase domain. To conclude, THE1 may be post-translationally modified in several ways, but the function of these post-translational modifications has not been tested.

## Evolutionary origin

To find the evolutionary origin of *Cr*RLK1L proteins we searched for putative *Cr*RLK1Ls proteins by BLASTing the Arabidopsis *Cr*RLK1Ls against a representative set of genomes from the plant kingdom (Altschul et al., 1990). This includes the following genomes: *Fragaria vesca* V4.0.a2 (Y., Li et al., 2019), *Solanum lycopersicum ITAG5.0* (Zhou et al., 2022), *Lactuca sativa* V8.0 (Reyes-Chin-Wo et al., 2017), *Ananas comosus* V3 (Ming et al., 2015), *Oryza sativa* V7.0 (Ouyang et al., 2007), *Cinnamomum kanehirae* V3 (Chaw et al., 2019), *Amborella trichopoda* V2.1 (Amborella Genome Project et al., 2013), *Picea abies* V1.0 (Nystedt et al., 2013), *Ceratopteris richardii* V2.1 (Marchant et al., 2022), *Diphasiastrum complanatum* V3.1 (Kuo et al., 2023), *Selaginella moellendorffii* V1.0 (Banks et al., 2011), *Marchantia polymorpha* V3.1 (Bowman et al., 2017), *Physcomitrium patens* V6.1 (Bi et al., 2024) and *Chara braunii* V1.0 (Nishiyama et al., 2018). All sequences were analyzed for the presence of a malectin-like domain (PFAM12819) and a kinase domain (PFAM07714/PFAM00069) in Geneious Prime V2025.1.3 using the InterPro V2.1 plugin (Table S2) (Blum et al., 2025; Paysan-Lafosse et al., 2025). We found a protein in the *Closterium peracerosum–strigosum–littorale* complex known as *Cp*RLK1 (Hirano et al., 2015), which has a similar domain composition as the *Cr*RLK1Ls and therefore included it in the analysis. We also found several genes with homology to the *Cr*RLK1Ls in the streptophyte alga *Chara braunii*, but closer inspection revealed an additional leucine-rich repeat domain in between the extracellular malectin-like domain and the transmembrane region. The average length of these algae proteins was found to be around 1.000 residues, whereas the length of the plant *Cr*RLK1Ls is between 800 and 900 residues (Table S2). The *Cr*RLK1Ls are believed to be a land plant specific gene family (Mecchia et al., 2022). Our findings align with the hypothesis that the *Cr*RLK1Ls are an invention specific to land plants. Probably, the colonization of land puts higher demands on the adaptability and strength of plant cell walls, due to increased fluctuations in water availability and different physical forces compared to life in water. The *Cr*RLK1Ls seem to function in pathways that allow adaptation to these challenges and may therefore have played a key role in land colonization by plants.

**Figure 2:**
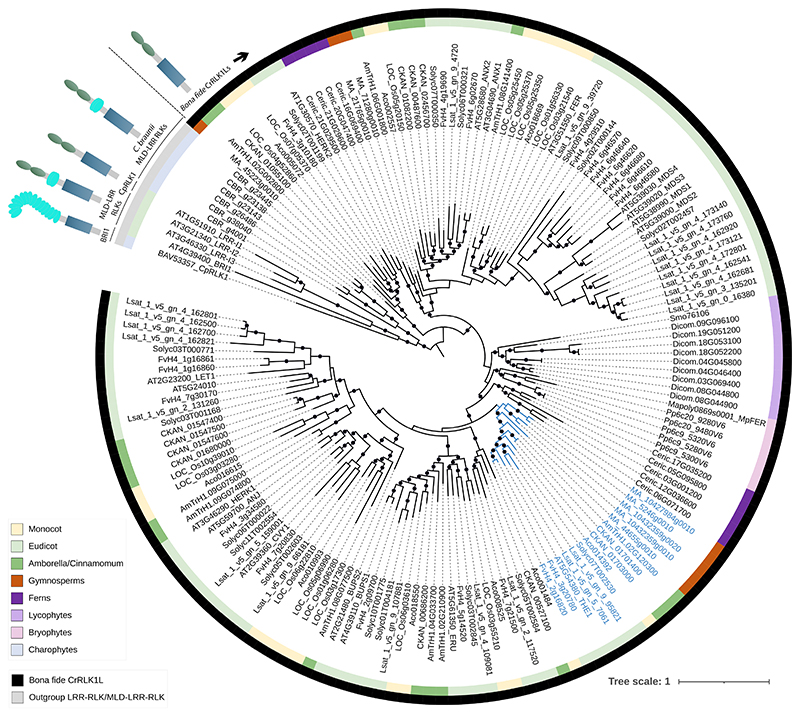
A sequence-based maximum-likelihood phylogeny of members of the *Catharanthus roseus* RECEPTOR-LIKE KINASE 1-LIKE (*Cr*RLK1L) family in different species. The branches and leaf labels of the clade containing THE1 are colored blue. The outer black and grey ring indicates which leaves correspond to *bona fide Cr*RLK1Ls versus the outgroup, while the inner colored ring shows the lineage of each leaf node. Branch support was assessed by 1000 bootstraps. Black dots on branches indicate bootstrap values over 90%. *Cr*RLK1Ls from the following species are included in the phylogeny: *Arabidopsis thaliana, Fragaria vesca, Solanum lycopersicum, Lactuca sativa, Ananas comosus, Oryza sativa, Cinnamomum kanehirae, Amborella trichopoda, Picea abies, Ceratopteris richardii, Diphasiastrum complanatum, Selaginella moellendorffii, Marchantia polymorpha*, and *Physcomitrium patens*. The sequences of the Arabidopsis receptor kinase BRASSINOSTEROID-INSENSITIVE1 (BRI1) and malectin-like domain- and leucine-rich repeat-containing receptor kinases (MLD-LRR RLKs) were included as an outgroup. We refer to Data S2 for the species and sequence of each node ID. Sequences were aligned with MAFFT V7 (algorithm FFT-NS-i) (Katoh & Standley, 2013), the alignment was trimmed with TrimAI V1.4 TrimAI (settings -gt 0.9 -cons 60) (Capella-Gutierrez et al., 2009), the phylogeny was inferred with IQ-TREE V2.0.3 (settings -m TEST -bb 1000 -alrt 1000) (Nguyen et al., 2015) and visualized in iTOL V7 (Letunic & Bork, 2024).

We created a sequence-based maximum-likelihood phylogeny of THE1 and related proteins to find the evolutionary origin of THE1 (Figure 2). We find that THE1 clusters in a clade containing *Cr*RLK1Ls from the gymnosperm *Picea abies*, a single *Cr*RLK1L from *Amborella trichopoda*, two members from *Cinnamomum kanehirae*, a single *Cr*RLK1L from *Ananas comosus*, and *Cr*RLK1Ls of several eudicot species. *Amborella trichopoda* and *Cinnamomum kanehirae* are angiosperm lineages distantly related to monocots and eudicots (Amborella Genome Project et al., 2013; Chaw et al., 2019; H.,-T., Li et al., 2019). Other studies found similar clustering of THE1 within a clade of *Cr*RLK1Ls including *Picea abies* and a single *CrRLK1L* member from *Amborella trichopoda* (Galindo-Trigo et al., 2016). The remaining *Cr*RLK1Ls from *Amborella trichopoda* clustered with other angiosperm *Cr*RLK1Ls than THE1, indicating that the angiosperm *Cr*RLK1L family already expanded before *Amborella trichopoda* diverged from its angiosperm sister lineages. The divergence of *Amborella trichopoda* from the other angiosperms is believed to have happened around 200 million years ago (H.,-T., Li et al., 2019). We therefore estimate that THE1 diverged from its angiosperm family members over 200 million years ago. No *Cr*RLK1Ls from monocots can be observed in the clade containing THE1 in our phylogeny. Other studies that compared *Cr*RLK1Ls from maize or wheat to *Cr*RLK1Ls from Arabidopsis do not find orthologs for THE1 either (Gawande & Sankaranarayanan, 2024; Wang et al., 2024). Notably, grass cell walls are more tolerant to ISX (Brabham et al., 2018). This is commonly explained by the major compositional difference between cell walls from grasses and dicots (Vogel, 2008; Brabham et al., 2018). For example, grasses have only 2-10% pectin in their primary cell walls, whereas the percentage of pectin is around 35% in the primary cell walls of dicot plant species (Mohnen, 2008; Vogel, 2008). However, the cellulose fraction is similar in grasses (20-30%) and dicots (15-30%) (Vogel, 2008). Perhaps, the absence of *THE1* also plays a role in the reduced response to ISX in grasses. Alternatively, the cell wall component monitored by THE1 may be absent or less essential in grasses, making THE1 dispensable.

We performed a multiple sequence alignment of several proteins within the THE1 clade and compared these sequences to Arabidopsis *Cr*RLK1Ls outside of the THE1 clade to find conserved and unique features in THE1 compared to the other *Cr*RLK1Ls (Figure S1-2). We found that the G37 residue in THE1 is conserved across all *Cr*RLK1Ls (Figure S2a). The G37 residue is mutated in *the1-1* and this leads to a loss of function(Hématy et al., 2007). When the corresponding glycine is mutated to a serine in FER it causes a temperature-sensitive phenotype (Kim et al., 2021). We detected two unique N-glycosylation sites at positions N154 and N168 for THE1 (Figure S2b). In addition, the kinase region is highly conserved between all *Cr*RLK1LS (Figure S2c). Finally, we observed that the C-terminal region of THE1 is conserved within the group of THE1 orthologs but not in *Cr*RLK1Ls outside the THE1-clade (Figure S2d). This C-terminal region contains several putative regulatory phosphorylation sites (Figure 1a, Table S1) (Hématy et al., 2007; Heazlewood et al., 2008). This may indicate that regulation of THE1-signaling is distinct from its *Cr*RLK1L family members. However, it has been shown that the intracellular domains of FER, ANX1 and HERCULES 1 (HERK1) are interchangeable with respect to their function in pollen tube reception, which supports the contrasting hypothesis that the ectodomains define *Cr*RLK1L specificity (Kessler et al., 2015).

## Stimulus perception

In this subchapter, we focus on the stimuli that are perceived by THE1. We define a stimulus as the change in the local environment that triggers a response. This is different from the signal, which we define as the ligand, physical force or energy that carries information about the stimulus and triggers a response. In the next subchapter, we elaborate on the signals THE1 perceives.

**Figure 3:**
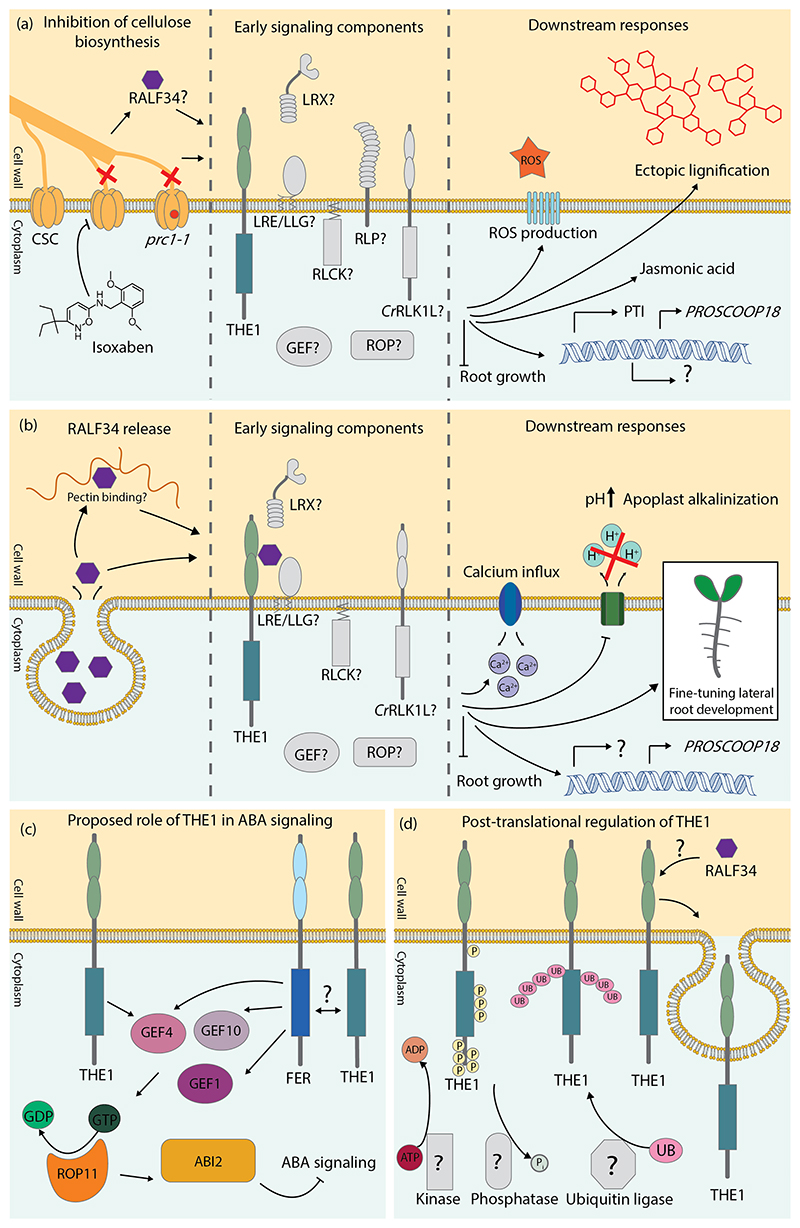
Schematic overview of the functions and regulation of THESEUS1 (THE1). (a) THE1 is involved in the response to the inhibition of cellulose biosynthesis. Cellulose biosynthesis takes place in cellulose synthase complexes (CSCs) and can be inhibited by mutations in the CELLULOSE SYNTHASE enzyme, like in the *procuste (prc1-1*) mutant, or by chemical inhibitors such as isoxaben. No co-receptors of THE1 have been described so far. Potential co-receptors of THE1 are LEUCINE-RICH REPEAT EXTENSINs (LRXs), the glycosylphosphatidylinositol-anchored proteins (GPI-AP) LORELEI (LRE) and LRE-like GPI-APs (LLGs), RECEPTOR-LIKE PROTEINs (RLPs), and other *Catharantus roseus* RECEPTOR-LIKE KINASE 1-LIKE (*Cr*RLK1L) proteins. Initial downstream components could be RECEPTOR-LIKE CYTOPLASMIC KINASES (RLCKs), GUANINE EXCHANGE FACTORs (GEFs), and RHO OF PLANTS (ROP). Responses to cellulose biosynthesis inhibition include production of reactive oxygen species (ROS), ectopic lignification, inhibition of root growth, jasmonic acid production, and the expression of genes involved in pattern-triggered immunity (PTI). (b) Responses mediated by THE1 in response to treatment with the RAPID ALKALINIZATION FACTOR 34 (RALF34) ligand. Little is known about when and how the RALF34 ligand is released. It is not known if RALF34 binds pectin like other RALFs. THE1 binds RALF34 with its ectodomain, which may involve co-receptors and could alter interactions with immediate downstream components. RALF34 treatment leads to calcium influx, apoplastic pH alkalinization, increased expression of *PROSCOOP18*, and inhibition of root growth. RALF34 and THE1 also play a role in the fine-tuning of lateral root development. (c) Hypothetical model of THE1 involvement in osmotic stress responses. The related *Cr*RLK1L FERONIA (FER) represses ABA signaling through GEF1/4/10, ROP11, and the ABI2 phosphatase. THE1 was shown to interact with GEF4, and this interaction may affect the ABA signaling pathway through GEF4. THE1 may also be involved in the pathway by directly interacting with FER. (d) Hypothetical modes of post-translational regulation of THE1. Regulation of THE1 may take place through phosphorylation, ubiquitination, or receptor internalization.

Many studies about THE1 focus on its role in the response to inhibition of cellulose biosynthesis (Figure 3a). The inhibition of cellulose biosynthesis has profound effects on plant physiology, which are not completely understood yet. Cellulose-deficient mutants, such as the *CELLULOSE SYNTHASE* 6 mutant *prc1-1*, are dwarfed and exhibit increased defense responses such as ectopic lignification and activation of jasmonic acid signaling pathways (Fagard et al., 2000; Caño-Delgado et al., 2003). In addition, *prc1-1* plants show a relative increase in hemicellulose levels and qualitative differences in pectin composition compared to wildtype plants (Fagard et al., 2000; Robert et al., 2004). Treatment with ISX removes cellulose synthase complexes from the plasma membrane within 20 minutes (Paredez et al., 2006). Subsequently, cell swelling and inhibition of cell elongation can be observed within the first 4 hours of ISX treatment (Refrégier et al., 2004; Hoffmann et al., 2024). At the molecular level, the response to ISX treatment shows many similarities to the immune response triggered by elicitors of pathogenic origin, and includes MITOGEN-ASSOCIATED PROTEIN KINASE activation, the production of reactive oxygen species (ROS), and expression of immune marker genes (Zhai et al., 2024; Chaudhary et al., 2020). Cellulose biosynthesis inhibition caused by defects in cellulose synthase subunits induce ectopic lignification throughout all tissues (Hématy et al., 2007), but the ectopic lignification remains restricted to growing cells in the case of ISX treatment (Merz et al., 2017). These observations show that cellulose biosynthesis inhibition activates a complex array of responses and shows that secondary cell wall components can be deposited in primary cell walls, indicating the existing plasticity of metabolic pathways.

THE1 was discovered in a suppressor screen of *prc1-1* (Fagard et al., 2000; Hématy et al., 2007). While dark-grown *prc1-1* mutant seedlings show strongly decreased hypocotyl elongation compared to wildtype plants, *prc1-1/the1* double mutants show an intermediate hypocotyl length (Hématy et al., 2007). THE1 is required for lignification and hormone production in response to inhibition of cellulose production by ISX (Denness et al., 2011; Merz et al., 2017; Engelsdorf et al., 2018). THE1 is also required for lignin deposition in Arabidopsis roots in response to tunicamycin, an inhibitor of N-linked glycan biosynthesis (Nakamura et al., 2022). This similarity might be explained by the fact that proper N-glycan processing is required for cellulose biosynthesis, since a S599F mutation in *RADIALLY SWOLLEN 3 (RSW3)*, encoding an N-glycan processing GLUCOSIDASE-II enzyme has reduced cellulose content (Peng et al., 2000; Burn et al., 2002; Nakamura et al., 2022). Taken together, inhibition of cellulose biosynthesis triggers THE1-dependent responses.

We discuss the role of THE1 in three processes that result from inhibition of cellulose biosynthesis: 1) altered activity of the cellulose synthase complexes, 2) a stimulus of mechanical origin and 3) a stimulus of chemical origin. A role in monitoring the activity of the cellulose synthase complexes may seem likely given the involvement of THE1 in the response to inhibition of cellulose biosynthesis. However, cellulose levels are unaltered in *the1-1* mutant (Hématy et al., 2007; Bacete et al., 2022), and internalization of cellulose synthase complexes following ISX treatment is not affected in the *the1-1* mutant either (Van der Does et al., 2017). Thus, there is no evidence so far for a direct role of THE1 in sensing and regulating the activity of cellulose synthase complexes. Another possibility is that THE1 is responsive to a mechanical stimulus that could be caused by cell wall distortion which follows cellulose biosynthesis inhibition and is visible as bulging of epidermal cells (Engelsdorf et al., 2018). There is limited understanding of the perception of mechanical stimuli in plants (Bacete & Hamann, 2020). A parsimonious model for the role of THE1 and other *Cr*RLK1Ls as mechanosensors would be that they act as a mechanical spring connecting the cell wall to the plasma membrane. However, a structural model supporting this hypothesis is still lacking. Alternatively, other molecular components might sense changes in mechanical properties and communicate this to THE1, which then transduces the information to the interior of the cell. A chemical stimulus could be a change in cell wall composition, cell wall structure, or cell wall pH. We expect that the inhibition of cellulose biosynthesis initially causes a reduced incorporation of cellulose in the wall. Yet, THE1 has not been shown to directly perceive changes in cellulose levels. The order of secondary effects, such as release of damage-associated molecular patterns or changes in the structure of cell wall components are notoriously difficult to study (Boerjan et al., 2024). The finding that loss of *THE1* restores apical hook formation in the pectin-deficient *quasimodo2-1* mutant indicates that *THE1* is responsive to changes in other cell wall components than cellulose alone (Lorrai et al., 2024). In addition, the root- and hypocotyl growth responses to nickel, lead, cadmium and zinc are affected in the *the1-6* and the *the1-4* mutant (Richter et al., 2017), suggesting that metal ions may also cause a stimulus to which THE1 is responsive. The THE1-dependent effects of heavy metals may also be traced back to alterations in pectin, since heavy metals can modify pectin structure (Wang et al., 2019). We hypothesize that THE1 is an integrator for both chemical and mechanical stimuli. This would involve other components of the CWI maintenance system sensing the stimuli, and subsequently directly interacting with THE1 or providing a signal that can regulate the activity of THE1. Such a mechanism could explain why THE1 is not only involved in the response cell wall damage but also in abiotic stimuli, such as hyperosmotic stress (Bacete et al., 2022).

## Signal perception and co-receptors

A milestone in THE1 research was the discovery of the RALF34 ligand (Gonneau et al., 2018). RALFs are small cysteine-rich peptide hormones named after their ability to cause alkalinization of plant cell culture medium and are involved in the regulation of growth, development, and stress responses (Pearce et al., 2001; Abarca et al., 2021). Gonneau et al., (2018) described three responses that follow RALF34 treatment: 1) RALF34 treatment leads to a calcium wave occurring within one minute after peptide application, which is absent in the *the1-1* mutant. 2) RALF34 treatment causes alkalinization of the extracellular space in a THE1-dependent manner. 3) RALF34 treatment leads to THE1-dependent inhibition of root growth. In addition, *the1-1, the1-4, the1-6, THE1*:*GFP* overexpressing lines, *RALF34* overexpression lines, and *ralf34* mutants displayed aberrant pericycle wall divisions and increased proportion of stage 1 lateral root primordia compared to wildtype plants (Figure 3b) (Murphy et al., 2016; Gonneau et al., 2018). The observation that both THE1 and RALF34 loss-of-function mutants and overexpression lines exhibit aberrant lateral root development suggests that proper dosage of either component is essential for normal lateral root development (Gonneau et al., 2018). RALF34 is not required for the lignification of root tips following ISX treatment (Gonneau et al., 2018). A question that remains is which stimulus triggers the release of the RALF34 signal. One proposed mechanism is that RALF34 is released from the cell wall upon cell wall damage occurring upon cellulose biosynthesis inhibition (Gonneau et al., 2018). Recently, an analogous model was proposed in which the FER ligand RALF22 regulates the growth of root hairs in a negative feedback loop involving the cell wall component pectin (Schoenaers et al., 2024). Briefly, RALF22 is secreted together with highly methylesterified pectin. Next, RALF22 bind to FER which leads to alkalinization of the apoplast and halts release of new cell wall material. Pectin methylesterases are more active at higher pH, which removes methyl groups from pectin and leads to a negative charge of the pectin backbone. Following this, positively charged RALF22 binds and compacts demethylesterified pectin. This depletes the reservoir of RALF22 available for FER, halting growth inhibitory activity by FER, and allowing the deposition of cell wall material to resume (Schoenaers et al., 2024). A similar mechanism could be possible for RALF34 and THE1. THE1 may also perceive other RALFs in addition to RALF34. The binding of more than one type of RALF is not uncommon in the *Cr*RLK1L family. To illustrate, RALF ligands of FER include RALF1, RALF23 and RALF33 (Haruta et al., 2014; Stegmann et al., 2017; Shen et al., 2025). Furthermore, THE1 could perceive other ligands in addition to RALFs, as several *Cr*RLK1L family members were described to bind pectin *in vitro* (Feng et al., 2018; Lin et al., 2022).

Additionally, THE1 probably interacts with co-receptors. The glycosylphosphatidylinositol-anchored protein (GPI-AP) LORELEI (LRE) and LRE-like GPI-APs (LLGs) act as co-receptors to *Cr*RLK1Ls in the perception of RALF peptides (Li et al., 2015; Ge et al., 2019; Xiao et al., 2019; Schoenaers et al., 2024). *LLG2* and *LLG3* are predominantly expressed in pollen tubes, whereas *LRE* and *LLG1* exhibit a broader expression pattern, including vegetative tissues such as leaves and seedlings (Wang et al., 2017; Ge et al., 2019). *THE1* is expressed at low or undetectable levels in pollen tubes (Lindner et al., 2012). This makes LRE and LLG1 the prime candidates in the LRE/LLG family to function as co-receptors for THE1. Another class of *Cr*RLK1L-interacting proteins is the LEUCINE-RICH REPEAT EXTENSIN (LRX) family (Baumberger et al., 2001; Herger et al., 2019). These proteins are anchored to the cell wall through their EXTENSIN domain (Fabrice et al., 2018). LRX proteins also directly interact with RALF peptides (Mecchia et al., 2017). Moreover, the LEUCINE-RICH REPEAT domain of LRX4 interacts with the extracellular domain of FER (Dünser et al., 2019). The LRXs may thus constitute a physical link between the cell wall and the *Cr*RLK1Ls, and convey information about the cell wall state through the *Cr*RLK1Ls to the interior of the cell (Herger et al., 2019; Manchanda & Geitmann, 2025). It is also proposed that *Cr*RLK1Ls form heteromeric complexes with each other (Schoenaers & Vissenberg, 2025). The extracellular domain of FER interacts with the extracellular domains of the *Cr*RLK1Ls HERK1 and ANJEA (Galindo-Trigo et al., 2020). THE1 may act in the same pathway as HERK1, because a *herk1-1/the1-4* double mutant has reduced petiole length, which was not observed in the single mutants (Guo et al., 2009). This interaction between *Cr*RLK1Ls may indicate that their signaling output is more complex than a one-ligand-one-receptor model, warranting study of *Cr*RLK1Ls signaling as a multi-component system, where multiple *Cr*RLK1Ls and RALFs together determine signaling output (Schoenaers and Vissenberg, 2025). The receptor-like protein RLP12 is expressed in the same region of the root as THE1 and is also required for modulating JA levels in response to ISX (Bacete et al., 2022). However, there are no accounts of physical interactions between RLPs and *Cr*RLK1Ls so far. RLPs might interact with other components of *Cr*RLK1L complexes, an example is the interaction of RLP53 with LLG1, which has been described to positively regulate immunity (Chen et al., 2022). To conclude, there are several candidate receptors and extracellular proteins which may cooperate with THE1, but their role as co-receptor awaits demonstration of functional interaction.

## Signal propagation

The canonical pathway of signal transduction upon ligand perception by RKs is receptor/co-receptor dimerization, phosphorylation of the kinase activation loop and phosphorylation of downstream targets (Galindo-Trigo et al., 2020; Bender & Zipfel, 2023). Downstream targets of *Cr*RLK1Ls include guanine nucleotide exchange factors (GEFs) and receptor-like cytoplasmic kinases (RLCKs) (Duan et al., 2010; Boisson-Dernier et al., 2015; Qu et al., 2017). THE1 directly interacts with GUANINE EXCHANGE FACTOR 4 (GEF4) which plays a role in the response to the fungal pathogen *Botrytis cinerea* (Qu et al., 2017). GEFs can activate GTP-hydrolyzing proteins of the RHO OF PLANTS (ROP) family, which act as molecular switches and signal transduction proteins (Denninger, 2024). There are 11 *ROPs* in the Arabidopsis genome which play roles in various responses such as growth, immunity and responses to abiotic stress (Li & Liu, 2012; Mulvey & Dolan, 2023; Denninger, 2024). The activity of ROPs can be decreased by GTPase ACTIVATING PROTEINs (GAPs) through promotion of the GTP-hydrolyzing activity of ROPs (Denninger, 2024). RLCKs are related to RKs but lack the extracellular and transmembrane domain (Hailemariam et al., 2024). They phosphorylate other components of the signaling pathway by phosphorylation, and some of their targets include RESPIRATORY BURST OXIDASE HOMOLOG D (RBOHD) and calcium channels, linking receptor activation and secondary responses, such as ROS production and intracellular calcium influx (Li et al., 2014; Ranf et al., 2014; Yu et al., 2019). Identification of GEFs other than GEF4, GAPs, ROPs, and RLCKs acting downstream of THE1 is still pending, yet crucial to understand early signaling events.

Interestingly, THE1 can activate downstream responses in the absence of its kinase domain. Merz et al. (2017) found that the *the1-4* allele (up until then believed to lead to the absence of a *THE1* transcript), encodes a truncated version of THE1 lacking most of the intracellular region. Plants containing the *the1-4* allele show enhanced responses to cellulose biosynthesis inhibition and the *the1-4* allele is therefore referred to as a hypermorphic allele (Merz et al., 2017). The *the1-3* mutant has a T-DNA insertion at a comparable location to the *the1-4* mutant (Figure 1a). However, an antisense effect was proposed to prevent expression of a transcript in the *the1-3* mutant, explaining why the *the1-3* mutant is generally considered a loss-of-function mutant (Merz et al., 2017). One example of the enhanced responses to cellulose biosynthesis inhibition in the *the1-4* mutant is a shorter root length in double mutants of *the1-4* and *cesa*^*3je5*^ cellulose-deficient mutant compared to the single *cesa3*^*je5*^ mutant (Pysh et al., 2012; Merz et al., 2017). Also, *the1-4* mutant plants exhibited enhanced JA production in response to ISX (Gigli-Bisceglia *et al*., 2018; Bacete *et al*., 2022). Therefore, it was hypothesized that the kinase domain is not required for initiating THE1-dependent responses but instead for dampening THE1 activity (Merz et al., 2017). As proposed before, this implies that the extracellular part of THE1 must interact with some other molecular component, which remain(s) to be identified (Merz et al., 2017). Ultimately, the absence of the kinase domain means that the *the1-4* version of THE1 lacks enzymatic activity and cannot directly activate intracellular components. Alternatively, the kinase domain might be required to inhibit the activity of other molecular components that normally induce responses to stimuli to which THE1 is responsive. In the absence of the kinase domain, this repressive action is lost, leading to enhanced responses (Merz et al., 2017).

Changes in cytoplasmic calcium levels, increase in apoplastic pH, and ROS production are known downstream responses of THE1 (Denness et al., 2011; Gonneau et al., 2018). ROS production after treatment of seedlings with ISX depends on a functional THE1 and also depends on the presence of *RBOHD* (Denness et al., 2011). A transient increase in intracellular calcium concentration and apoplast alkalinization occur after treatment with RALF34 and depend on THE1 (Gonneau et al., 2018). Moreover, the signaling pathways of THE1 and FER overlap on calcium influx, since pre-treatment with RALF1 (the ligand of FER), prevents a subsequent calcium influx after RALF34 treatment, and vice versa (Gonneau et al., 2018).

An underexplored topic is how THE1 affects cytoskeletal organization. Cell wall components and enzymes responsible for the biosynthesis of novel cell wall components at the plasma membrane are delivered to the cell wall via vesicle trafficking from the Golgi apparatus (Cosgrove, 2024b). This vesicle trafficking from Golgi to the plasma membrane happens along actin filaments (Richter et al., 2009). ISX treatment slows the movement of Golgi bodies and also reduces actin filament remodeling (Hoffmann et al., 2024). The role of THE1 in regulating actin dynamics is not known but potentially relevant. The dynamics of microtubules, another major cytoskeleton component, can be altered by motor proteins, one of which is KINESIN-13a (Lu et al., 2005; Oda & Fukuda, 2013). A loss-of-function mutant for *KINESIN-13a* shows increased petal length (Fujikura et al., 2014). Interestingly, petal length is restored to wildtype levels in the *kinesin-13a*/*the1-4* double mutant, suggesting that THE1 influences microtubule dynamics (Fujikura et al., 2014). The calcium spike that follows THE1 activation by RALF34 treatment may link THE1 to cytoskeleton dynamics, since both microtubule and actin dynamics are affected by changes in intracellular calcium concentration (Qin et al., 2012; Qian & Xiang, 2019) However, it remains to be determined how direct or indirect THE1 influences cytoskeleton organization.

THE1 furthermore regulates the expression of *PROSCOOP18*, a gene encoding the precursor of a small peptide of the SERINE RICH ENDOGENOUS PEPTIDE (SCOOP) family involved in immune responses (Zhai et al., 2024). Both treatment with RALF34 and ISX lead to increased expression of *PROSCOOP18* (Zhai et al., 2024). SCOOP peptides are ligands for the RK MALE DISCOVERER 1-INTERACTING RECEPTOR LIKE KINASE 2 (MIK2) (Rhodes et al., 2021; Yang et al., 2023). Disruption of MIK2 function strongly attenuates ISX-induced responses including immune marker gene expression, JA biosynthesis, and lignin deposition, reminiscent of the *the1-1* mutant phenotype in response to the same treatment (Van der Does et al., 2017). The MIK2-SCOOP18 module appears to act downstream of THE1, because expression levels of *PROSCOOP18* following ISX or RALF34 treatment are reduced in the *the1-1* background (Zhai et al., 2024).

THE1 modulates the expression of several more genes. The transcription factor *WRKY DNA-BINDING PROTEIN 40* and putative ubiquitin ligase *XB3 ORTHOLOG 4 IN ARABIDOPSIS THALIANA* are upregulated in a THE1-dependent manner after RALF34 treatment (Gonneau et al., 2018). An increase in the expression of the stress-related genes *CINNAMOYL COA REDUCTASE 1, PLANT DEFENSIN 1.2A, CYTOCHROME P450 FAMILY 81 SUBFAMILY F POLYPEPTIDE 2, FLG22-INDUCED RECEPTOR-LIKE KINASE 1*, and *RBOHD* in response to ISX also depends on THE1 (Chaudhary et al., 2020). No direct interactions between THE1 and transcription factors have been shown yet. Conversely, FER is known to phosphorylate the transcription factor and master regulator of JA-signaling MYC2 (Guo et al., 2018). FER also negatively regulates ABA signaling by phosphorylating and destabilizing the transcription factor ABA INSENSITIVE5 (ABI5) (Wang et al., 2022). We hypothesize that THE1 also interacts with transcription factors to determine downstream responses.

THE1 regulates the production of the plant stress hormones JA and ABA. Plants with the hypermorphic *the1-4* allele show increased JA levels upon treatment with ISX or Driselase (a cocktail of cell wall degrading enzymes) (Engelsdorf et al., 2018; Bacete et al., 2022). On the contrary, *the1-1* loss-of-function mutant plants have lower JA levels compared to the wildtype after cellulose biosynthesis inhibition or Driselase treatment (Engelsdorf et al., 2018; Bacete et al., 2022). The role for THE1 in ABA signaling and other ABA-associated pathways has only been discovered recently (Bacete et al., 2022). Analysis of hormone levels demonstrated that ABA accumulates to higher levels in *the1-1* mutant seedlings than in wildtype seedlings upon hyperosmotic treatment (Bacete et al., 2022). In addition, leaves of the *the1-1* mutant are more prone to losing turgor pressure and have slightly increased stomatal conductance (Bacete et al., 2022). Among the *Cr*RLK1Ls, *THE1* stands out among the *CrRLK1Ls* because its expression in leaves is downregulated in response to abiotic stresses more than that of all other family members (Lindner et al., 2012). This aligns with its proposed role as a negative regulator of ABA levels (Bacete et al., 2022).

The opposite effects of THE1 on ABA and JA production led Bacete et al. (2022) to propose a model in which cell wall damage activates THE1 and leads to JA production. Conversely, according to this model hyperosmotic shock alters the plasma membrane–cell wall continuum in such a way that THE1 becomes deactivated (possibly by transcriptional regulation), thereby lifting the repression of ABA signaling. In another pathway, FER represses ABA signaling through the GEF1/4/10–ROP11 pathway (Figure 3c) (Yu et al., 2012). In this pathway, the active GTP-bound conformation of ROP11 is induced by GEF1, GEF4 or GEF10 (Yu et al., 2012). Then, ROP11 interacts with the ABI2 phosphatase, which enhances its activity. ABI2 dephosphorylates downstream targets to repress ABA signaling (Merlot et al., 2001; Cutler et al., 2010). THE1 interacts with GEF4 to determine the response to fungal invasion (Qu et al., 2017). It may also interact with GEF4 to influence ABA signaling, possibly overlapping with FER in the GEF1/4/10-ROP11 pathway. However, a key difference between FER and THE1 is that THE1 acts as a positive regulator of JA, whereas FER acts as a negative regulator of JA (Engelsdorf et al., 2018; Guo et al., 2018; Zhao et al., 2021; Bacete et al., 2022). We currently do not know whether THE1 and FER interact physically, nor how this putative interaction could affect downstream responses. It also remains unclear how THE1 is post-translationally regulated during the signaling process. Elucidating the pathways and players involved will provide important insights into the regulation of plant hormone production.

## Influence of THE1 on cell wall structure and dynamics

The mechanical properties of the cell wall are influenced by polymer composition and structure (Cosgrove, 2022). Here we discuss the relation between THE1 and cell wall mechanics. Root cell walls of *the1-1* mutant plants have reduced stiffness as measured by Brillouin microscopy, a contact-free and *in vivo* method determining cell wall stiffness and viscosity, indicating that *THE1* plays a role in determining mechanical properties of the cell (Bacete et al., 2022). As the relative composition of the major carbohydrate components in the cell wall does not differ between *the1-1* and wildtype Arabidopsis in control conditions (Van der Does et al., 2017; Bacete et al., 2022), the difference in mechanical properties observed in *the1-1* plants is more likely the result of changes in cell wall structure and the activity of cell wall modifying enzymes. We want to emphasize the role of three THE1-linked components in determining mechanical properties of the cell wall: the expansin proteins, the aromatic polymer lignin and the carbohydrate polymer pectin.

The α-expansins (EXPAs) are cell wall modifying enzymes capable of loosening the structure of the cell wall by non-enzymatically interacting with cellulose microfibrils (Cosgrove, 2024a). Interestingly, *EXPA1* was found to be downregulated in the *prc1-1/the1-1* double mutant compared to the *prc1-1* single mutant (Hématy et al., 2007). Increased cell wall stiffness was observed three hours after induction of *EXPA1* in (Samalova et al., 2023). Lower *EXPA1* expression may result in reduced cell wall stiffness in the *prc1-1*/*the1-1* double mutant compared to the *prc1-1* single mutant, potentially leading to increased cell elongation observed in the double mutant. It may seem counterintuitive that overexpression of a “cell wall loosening enzyme” causes increased cell wall stiffness. Yet, the overexpression of *EXPA1* was accompanied by changes in the expression of several other *EXPAs* and cell wall-modifying enzymes, such as *XYLOGLUCAN:XYLOGLUCOSYL TRANSFERASEs* (Samalova et al., 2023). The observed increase in stiffness may thus be a secondary effect or the consequence of CWI being activated.

Lignin is a phenolic polymer, which is deposited in secondary cell walls, where it waterproofs xylem and provides structural support by stiffening the cell wall, but it is generally absent from primary cell walls (Barros et al., 2015). It can be ectopically deposited in stress conditions to prevent pathogen invasion, seal wounds, or limit water diffusion (Barros et al., 2015; Lee et al., 2019). Ectopic lignification also occurs in cellulose deficient mutants or after ISX treatment (Hématy et al., 2007; Denness et al., 2011). This ectopic lignin may be a compensatory measure for the loss of cellulose or be part of the defense response. Loss-of-function mutants of THE1 display a strong reduction in ectopic lignin production under cellulose biosynthesis inhibition (Hématy et al., 2007; Denness et al., 2011; Merz et al., 2017). This indicates that THE1 modulates ectopic lignification. However, no major changes in lignin content have been described for *the1* mutants in control conditions (Hématy et al., 2007; Merz et al., 2017). It therefore appears unlikely that changes in cell wall lignin content are the reason for reduced stiffness in *the1-1* roots in control conditions.

Pectin is a structurally complex and versatile carbohydrate polymer (Mohnen, 2008). Its structure and properties can be modified by enzymatic activity, pH and the presence/absence of ions or ligands (Delmer et al., 2024; Schoenaers et al., 2024; Obomighie et al., 2025). The addition of calcium ions to de-methylesterified pectin increases the stiffness of pectin gels by crosslinking pectin backbones (Ngouémazong et al., 2012). In addition, the secretion of RALF peptides is described to compact and increase stiffness of de-methylesterified pectin, indicating that RALFs play roles both in cell wall signaling and as a structural component of the cell wall (Moussu et al., 2023; Schoenaers et al., 2024). The *Cr*RLK1Ls play a major role in determining this pectin structure, since they modify apoplastic pH in response to RALF binding, thereby determining the activity of pectin-modifying enzymes and charge of the pectin polymer (Haruta et al., 2014; Schoenaers & Vissenberg, 2025). Not much is known yet about the influence of THE1 on pectin structure or the role of pectin in determining THE1 activity. However, recently it was discovered that loss of *THE1* rescues apical hook formation in the pectin-deficient *quasimodo2-1* mutant (Mouille et al., 2007; Lorrai et al., 2024). Further investigation into the relationship between THE1 and pectin is likely to provide valuable information about THE1 functioning.

We want to emphasize that there are many direct and indirect routes by which the mechanical properties of cell walls can be altered. It is not known how the altered stiffness in the *the1-1* mutant is established. Understanding how cell wall components and enzymes interact to shape mechanical properties will be a formidable challenge to come. Novel cutting-edge tools such as Brillouin microscopy and molecular probes, however, provide us with unprecedented capability to measure mechanical properties of cell walls *in vivo* in the future (Michels et al., 2020; Alonso Baez & Bacete, 2023; Besten et al., 2025).

## Conclusion

Almost twenty years after its discovery, we find that there are still many open questions about THE1 remaining to be answered. THE1 has been implicated in several stress responses, including cell wall modification and hormone signaling. However, the mechanisms leading from stimuli to these responses through THE1 are largely unknown. This makes THE1 a promising and interesting topic of study, especially given the deep conservation of *THE1* in the angiosperm lineage. Both loss- and gain-of-function alleles are described. In the future, these can be supplemented with mutant lines encoding kinase-dead versions of THE1, variants mutated in N-glycosylation sites or variants in which domains are swapped with those of other *Cr*RLK1Ls. Studies with such mutant lines will further disentangle the mechanism by which THE1 functions and what differentiates it from other *Cr*RLK1Ls. Moreover, it would be valuable to learn which components act upstream of THE1 and/or release of its RALF34 ligand. Analysis of the promotor region may lead to identification of upstream transcriptional regulators. An ideal starting point for further functional characterization of THE1 would be the identification and validation of THE1 interactors, for example by protein pulldown experiments or proximity labeling (Qin et al., 2021; Yilmazer et al., 2023), which will enable the mapping of the molecular pathways in which THE1 functions. Transcriptomic experiments can be performed to identify how the transcriptome is affected in different *the1* mutants. Novel techniques for mapping mechanical properties such as Brillouin microscopy and molecular rotors can be used to show *in vivo* how cell wall properties are affected in *the* mutants (Alonso Baez & Bacete, 2023; Besten et al., 2025). Most importantly, shining light on the knowledge gaps in THE1-based signaling can provide insights into the role of THE1 in CWI maintenance. This could aid the knowledge-based development of resilient plant varieties required to safeguard plant performance in the face of stresses induced by ongoing climatic and environmental changes.

## Supplementary Material

Supplementary Material

Supplementary Figure and Table Legends

## Data Availability

All the article's supporting data and materials can be accessed in the supplementary information of the article.

## Box 1 main points

- *THE1* diverged from the other angiosperm *CrRLK1L* receptor kinases over 200 million years ago.
- THE1 is the first member of the *Cr*RLK1L family characterized as a component of the plant cell wall integrity maintenance mechanism in *Arabidopsis*.
- One ligand (RALF34) and one intracellular interaction partner (GEF4) of THE1 have been identified.
- THE1 modulates cell wall stiffness, ABA and JA production, suppresses cell elongation in response to cellulose biosynthesis inhibition, and is required for stress-induced lignin deposition.
- The kinase domain of THE1 is not required for activation of responses to cellulose biosynthesis inhibition.

## Box 2 Open questions and perspectives

- Which stimuli are activating THE1?
- How is the RALF34 ligand regulated and released?
- Which molecular components function upstream and downstream of THE1?
- What is the function of the kinase domain of THE1?
- How is THE1 involved in the modulation of the mechanical properties of the cell wall and CWI?

## Significance statement

THESEUS1 (THE1) is a receptor kinase for which many mutant phenotypes were described in relation to growth and stress responses in Arabidopsis. However, early signaling events by which these responses are established remain largely unexplored. Here we reveal the evolutionary origin of THE1, discuss what is unknown about THE1-based signaling and propose methods to address this knowledge gap.
