# Supplementary material for: The function of the Arabidopsis receptor kinase THESEUS1 in plant cell wall integrity maintenance: from evolutionary origin to future perspectives
Source: Plant J. Author manuscript; Available in PMC 2026 Mar 15. (PMC7618880; doi:10.1111/tpj.70701)
Supplement: Supplementary Material [file EMS212342-supplement-Supplementary_Material.zip › tpj70701-sup-0002-figures2.pdf]

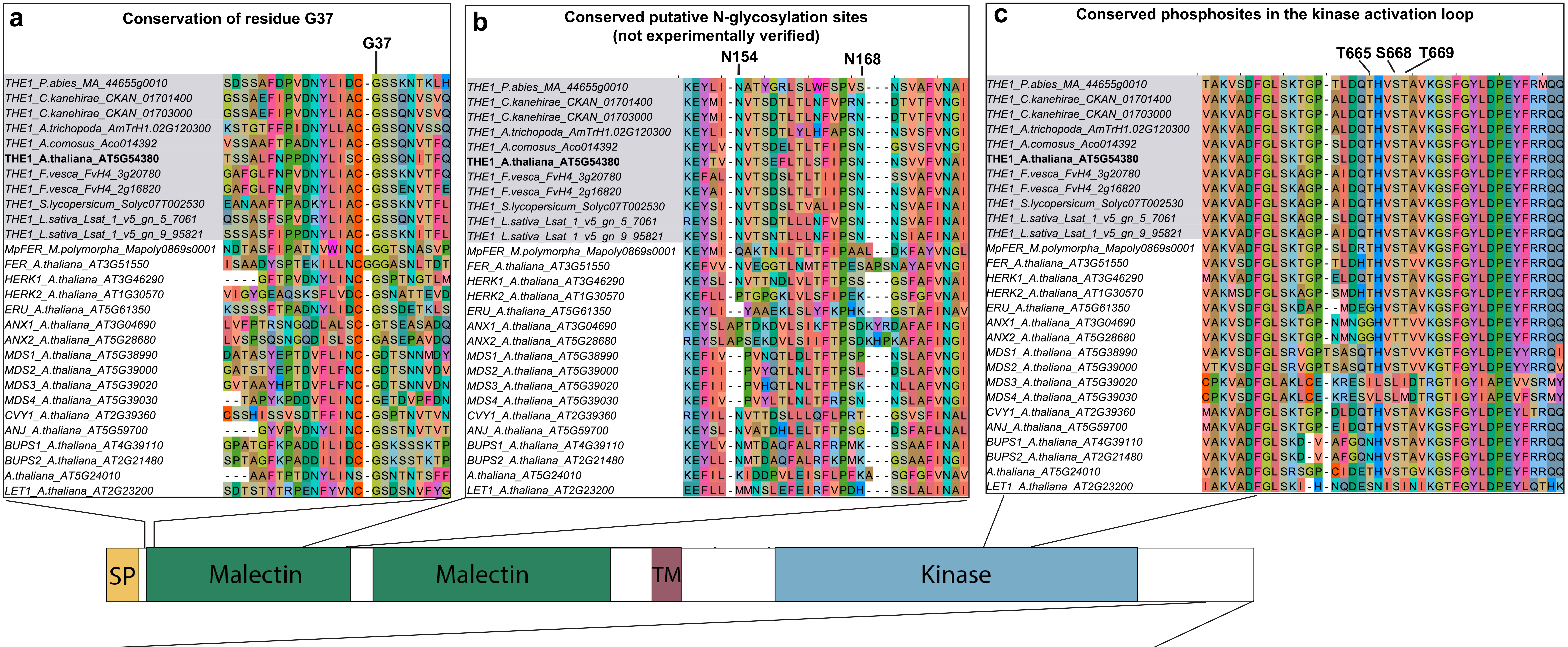

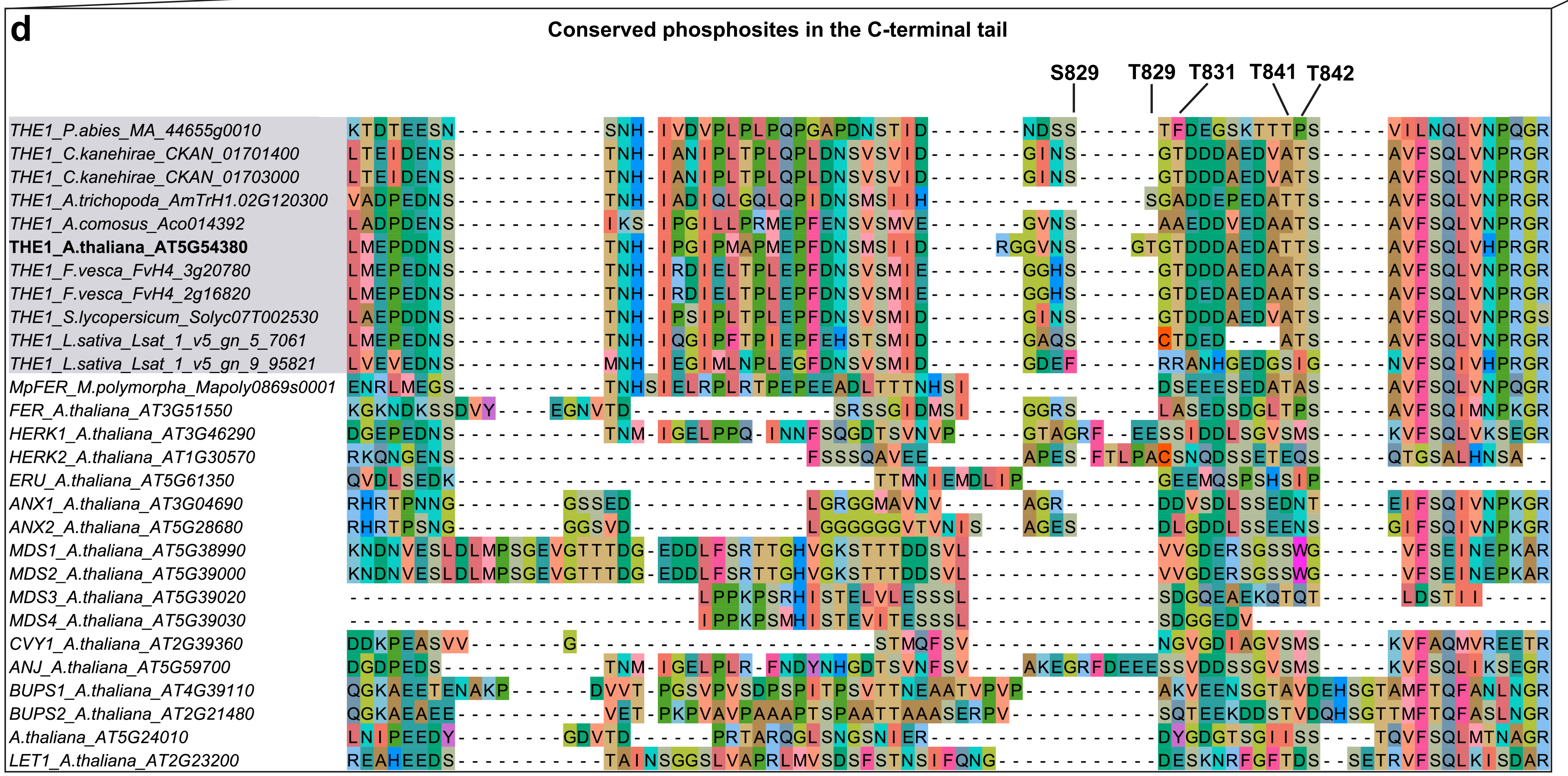

Figure S2 Sequence comparison between THESEUS1 (THE1) orthologs and other *Catharanthus roseus* RECEPTOR-LIKE KINASE 1-LIKE (*Cr*RLK1L) proteins. THE1 orthologs, the single *Marchantia polymorpha Cr*RLK1L *Mp*FER, and *Arabidopsis thaliana CrRLK1Ls* outside of the THE1 clade were aligned with MAFFT V7 (algorithm FFT-NS-i) and visualized in Jalview V2 (Waterhouse et al., 2009; Katoh and Standley, 2013). The residue numbering above the alignment indicates the position in the alignment relative to the THE1 sequence of *Arabidopsis thaliana*, of which the sequence name is indicated in bold. The sequence labels for THE1 orthologs are indicated by a grey background. (a) G37 is conserved across the *Cr*RLK1L family. (b) Two THE1-specific N-glycosylation motifs (N-X-S/T) can be found at positions 154 and 168 of THE1 (c). These N-glycosylation sites have not been experimentally verified. The kinase region is another strongly conserved region in the *Cr*RLK1L family. Here, the kinase activation loop is depicted, and THE1 phosphorylation sites at positions T665, Ser668, and T669 are indicated. (d) The C-terminal region is conserved within THE1 orthologs. Putative THE1 phosphorylation sites at positions S829, T831, T833, T841 and T842 are indicated. The phosphorylation sites depicted in c and d are based on data from the PhosPhAt 4.0 database (Table S1) (Heazlewood et al., 2008).
