# Supplementary figures and images for: The function of the Arabidopsis receptor kinase THESEUS1 in plant cell wall integrity maintenance: from evolutionary origin to future perspectives
Source: Plant J. Author manuscript; Available in PMC 2026 Mar 15. (PMC7618880; doi:10.1111/tpj.70701)

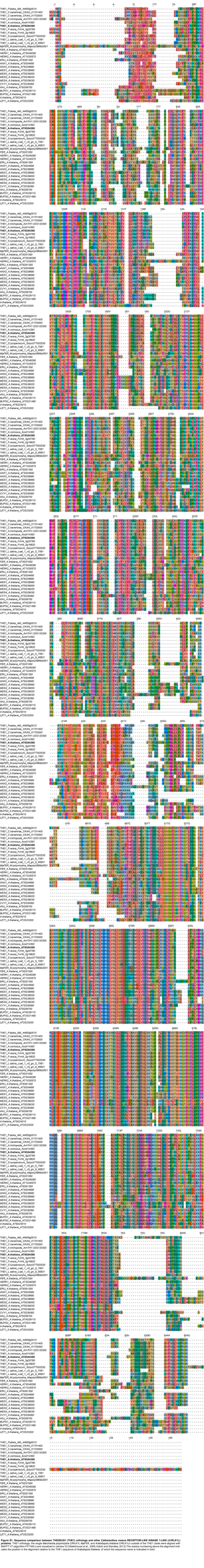

Supplement: Supplementary Material [file EMS212342-supplement-Supplementary_Material.zip › tpj70701-sup-0001-figures1.pdf]
