# Supplementary material for: The function of the Arabidopsis receptor kinase THESEUS1 in plant cell wall integrity maintenance: from evolutionary origin to future perspectives
Source: Plant J. Author manuscript; Available in PMC 2026 Mar 15. (PMC7618880; doi:10.1111/tpj.70701)
Supplement: Supplementary Figure and Table Legends [file EMS212342-supplement-Supplementary_Figure_and_Table_Legends.pdf]

Supplementary information

Figure S1. Multiple sequence alignment comparing THE1 homologs in different species to other *Cr*RLK1Ls.

Figure S2. Sequence comparison between THE1 and other *Cr*RLK1Ls highlighting conserved post-translational modification sites.

Table S1. THE1 phosphosites retrieved from PhosPhAt 4.0 Arabidopsis protein phosphorylation site database.

Table S2. Protein sequences used in this study.
